# Differences in the rates of admission and major orthopedic surgery care between Turkish and displaced children injured in a major earthquake

**DOI:** 10.1186/s13049-024-01286-y

**Published:** 2024-11-11

**Authors:** Mehmet Cenk Belibağlı, Mehmet Yiğit Gökmen, Ahmet Pamiry

**Affiliations:** 1 Institute of Medical Sciences, Cukurova University, Adana, Türkiye; 2 Department of Orthopaedics and Traumatology, University of Health Sciences, Adana City Training and Research Hospital, Adana, Türkiye; 3 Department of Orthopaedics and Traumatology, Yüreğir State Hospital, Adana, Türkiye

**Keywords:** Earthquake, Refugees, Immigrants, Children, Crush injuries, Fracture, Bone, Surgical treatments, Disaster medicine

## Abstract

**Background:**

The burden of displacement makes child refugees, asylum seekers, and migrant children less resistant to medical problems. On February 6, 2023, the southeast and the southcentral regions of Türkiye were struck by two sequential large earthquakes. The study aimed to analyze the characteristics of musculoskeletal injuries and the initial orthopedic surgery data resulting from the earthquake and compare the differences between Turkish and displaced children, particularly those who underwent major musculoskeletal interventions at the four state hospitals of the Adana metropolitan region.

**Methods:**

The retrospective study analyzed the medical records of the survivors admitted to the four general hospitals run by the government in the Adana, Türkiye metropolitan area between February 06, 2023, and February 13, 2023, the first seven days following the earthquake. The analysis data included age, gender, nationality, time of admission, types and localization of injuries, and treatment methods.

**Results:**

The number of cases under 18 years of age requiring orthopedic intervention was 969. The distribution of the cases based on nationality was as follows: there were 517 Turkish (53.35%), 371 Syrian (32.71%), and 81 children of unknown nationality (CUN) (8.36%). The files show that the patients requiring surgery, including fasciotomy, fracture surgery, and amputation (*n* = 281), were mainly treated at the level I trauma center (*n* = 171, 60.85%). In terms of the daily distribution of admissions based on the type of musculoskeletal injuries, the admissions of children with foot fractures in the first days were significantly increased (*p* = 0.0134). Regarding surgery rates, the fracture surgery and fasciotomy rates were significantly higher in cases admitted earlier. (*p* < 0.0001 and *p* = 0.0009, respectively). In terms of nationality, there were no significant differences regarding the date of admission, the number of cases who underwent amputations, and the discharge number and types.

**Conclusion:**

The study revealed that after the severe earthquake disaster, the state hospitals of the Adana metropolitan region provided unbiased healthcare for all the region’s children. Regarding orthopedic care, the response was given accordingly in this particular disaster, highlighting that level I trauma centers should always be ready for such intensity due to the short preparation time in case of a severe disaster.

## Introduction

In the last decades, the rate of displacement of people increased gradually. The records of the Population Division of the United Nations Department of Economic and Social Affairs show that in 2021 the number of international migrants was approximately 281 million, accounting for 3.5% of the world population, which was 2.8% two decades ago, and 2.3% in 1980 [1]. The demographics in the report of the United Nations High Commissioner for Refugees stated that the percentage of the displaced who were under 18 years old was 40% [2]. Türkiye has also played its part in this global migration environment. In the aftermath of the 2011 conflict in Syria on Türkiye’s southern border, there has been a rapid and intense influx of refugees into Türkiye [3]. The official numbers indicate that 1.3% of displaced persons live in camps. The remaining 98.7% was distributed all over Türkiye. As of July 18, 2024, the number of foreigners under temporary protection living in cities was 3,107,380, and approximately 1,561,640 were under the age of 18. The city of Adana, located in the southcentral region (eastern Mediterranean) with a population of more than 2.3 million, based on both the ratio of foreigners under temporary protection to the province’s population and the total number of foreigners, was the fifth city by hosting 218,369 people [4].

The burden of displacement makes child refugees, asylum seekers, and migrant children less resistant to medical problems [5]. Although numerous research studies have identified the medical conditions of this particular group, studies assessing the fulfillment of their healthcare needs are scarce [6]. In addition to congested living conditions with low sanitation, the restrictive measures of the receiving states impair the quality of life by reducing access to basic needs such as healthcare and education [7].

On February 6, 2023, the southeast and the southcentral regions of Türkiye were struck by two sequential large earthquakes nine hours apart with magnitudes of 7.8 Mw and 7.6 Mw, respectively [8]. As a result of the disaster affecting approximately 15 million people, 50,783 lost their lives, and 107,204 were injured. The official reports state that the total number of buildings categorized as collapsed or severely damaged was 518,009. The number of moderately damaged dwellings was estimated as 131,577, leaving over 2 million people directly facing housing problems [9]. In the Adana province after the second quake, the number of dwellings that were inhabitable due to having collapsed or had heavy damage was 2,952, in addition to nearly a hundred thousand with moderate or minor damages. The reports indicate that the level of damage in all 23 s- and third-level healthcare facilities owned by private and run by the government in the Adana Metropolitan area was low or none at all [10]. Among the 23, there were only four general hospitals run by the government, each located in one district, out of which one was a teaching hospital equipped with a level I trauma center.

The reports show that the most common types of musculoskeletal injuries suffered by children who survived an earthquake were fractures and wounds, and soft tissue injuries related to fractures and extremity crush injuries [11, 12].

The study aimed to analyze the characteristics of musculoskeletal injuries and the initial orthopedic surgery data resulting from the earthquake and compare the differences between Turkish and displaced children particularly those who underwent major musculoskeletal interventions at the four state hospitals of the Adana metropolitan region.

## Methods

The retrospective study analyzed the medical records of the survivors admitted to the general hospitals run by the government in the city of Adana metropolitan area between February 06, 2023, and February 13, 2023, the first seven days following the earthquake. The data was collected from four hospitals: Seyhan State Hospital, Yüreğir State Hospital, Çukurova State Hospital, and Adana City Training and Research Hospital. The latter was the only facility involving a level I trauma center, while the other three included level II centers.

The analysis data included age, gender, nationality, time of admission, types and localization of injuries, and treatment methods. The files that did not include the diagnostic International Classification of Diseases (ICD) codes X34: Victim of cataclysmic earth movements caused by earthquake and X39: Exposure to forces of nature, and were not treated or consulted by the orthopedics clinic were excluded.

The musculoskeletal injuries were classified into three groups: fracture surgeries (large bone fractures, including the pelvic ring), fasciotomies, and large bone amputations and joint disarticulations.

The discharge information was recorded as treated, transferred, hospitalized, and deceased.

**Fig. 1:**
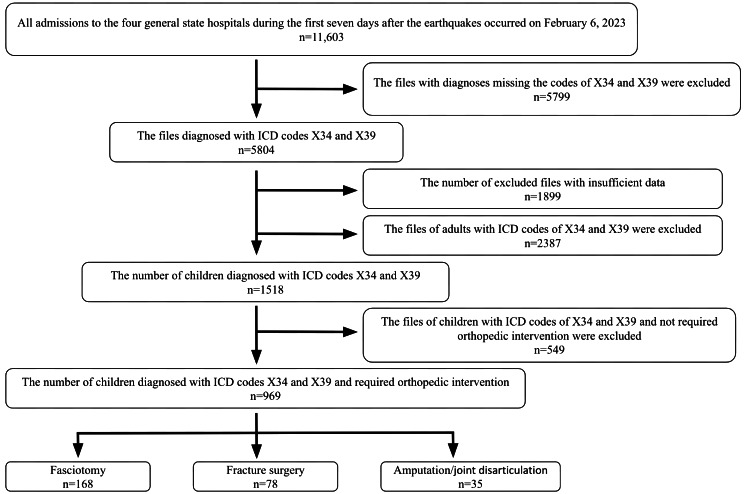
The study flowchart

The study flowchart is presented in Fig. 1.

### Ethical approval

The ethical approval of the study was given by the Clinical Research Ethics Committee of the Adana City Training and Research Hospital on December 7, 2023, with decision no: 2980. Written informed consent is not applicable.

### Statistics

The statistical analyses were performed using InVivoStat 4.10, UK. Descriptive statistics were reported as numbers, percentages, mean, and standard deviation. The numbers and percentages were rounded to the nearest value. Kolmogorov-Smirnov and Shapiro-Wilk tests were performed for categorical variables and were compared using ANOVA or Kruskall-Wallis tests according to the conformity to normal distribution. Parameters with significance in numerical variables were compared using unpaired t-tests or Mann-Whitney U tests according to the conformity for normal distribution. The Pearson and the Spearman tests were used for correlation analysis. The results were evaluated at a 95% confidence interval, and *p* < 0.05 was considered significant.

## Results

In the first seven days after the earthquakes, a total of 11,603 patients were admitted to emergency departments. There were 5804 (50.02%) cases diagnosed with X34 and X39. The number of cases under 18 years of age requiring orthopedic intervention was 969. During admission, the nationality information of the children was based on the identification cards (ID), and for the ones missing the IDs, the information provided by the rescuers or the accompanying person’s declaration was taken into account. The distribution of the cases based on nationality was as follows: there were 517 Turkish (53.35%), 371 Syrian (32.71%), and 81 children of unknown nationality (CUN) (8.36%). The researchers agree on two facts that the CUN should not be in the group of displaced children, almost all the Syrian population in the area were accepted as displaced persons, and the children with Syrian nationality were considered as displaced children in the interpretation of the study results.

**Fig. 2:**
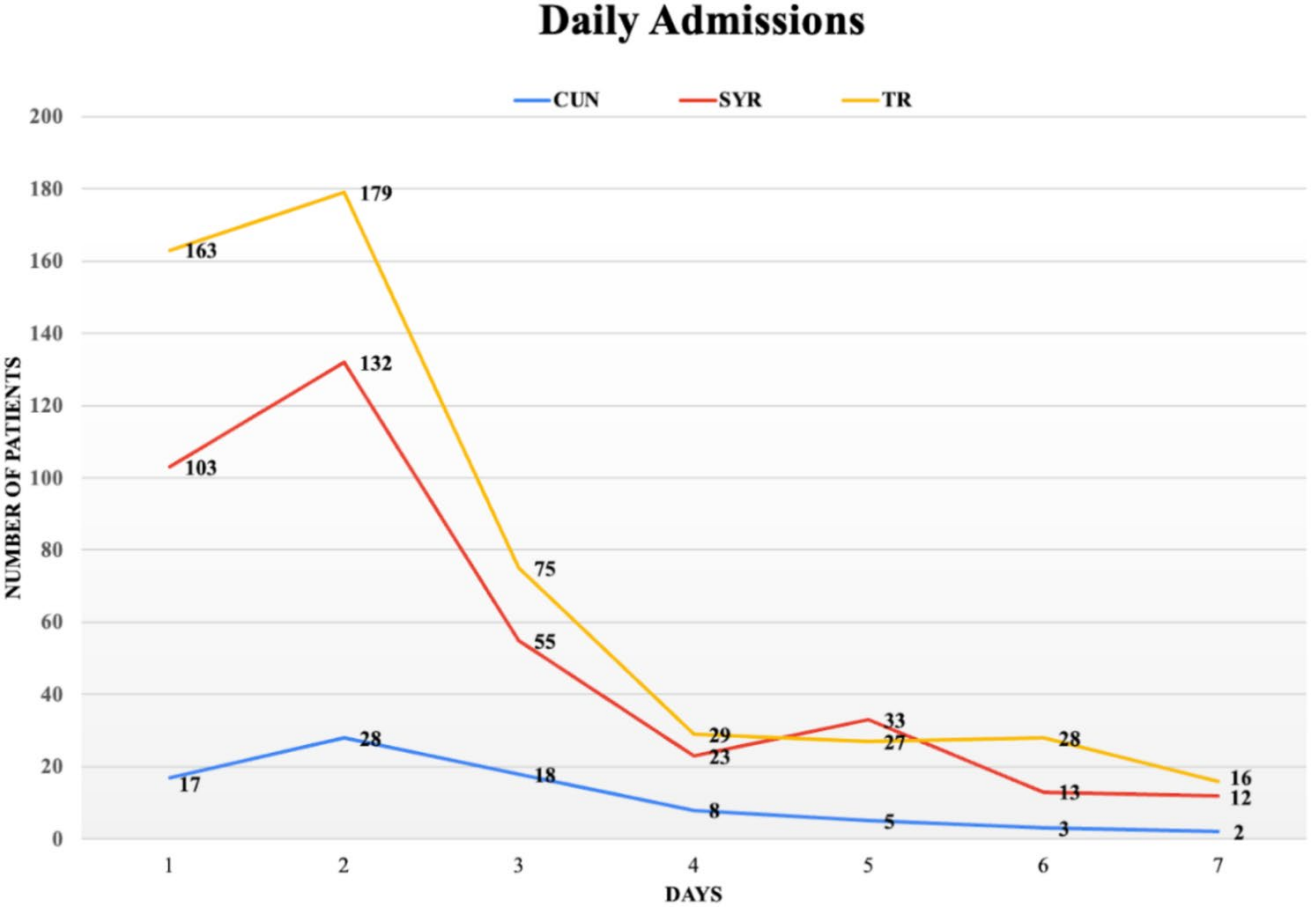
Daily admissions to hospital emergency departments by nationality in the first week after the earthquake

The majority of the cases underwent closed reduction, splinting, bandaging, and additional conservative fracture treatment (*n* = 688, 71%). The files show that the patients requiring surgery, including fasciotomy, fracture surgery, and amputation (*n* = 281), were mainly treated at the level I trauma center (*n* = 171, 60.85%). The number of daily admissions of children peaked on the second day and gradually increased (Fig. 2). The daily number of admissions in the first seven days after the earthquake were 283, 339, 148, 60, 65, 44, and 30, respectively.

The medical records noted on the 7th day indicate that 167 (17.23%) of the children were hospitalized, 588 (60.68%) were treated and discharged, 188 (19.40%) were transferred to hospitals in the cities not struck by the quakes, and 26 (2.68%) died. The demographics and major clinical intervention data are presented in Table 1.

**Table 1:** The basic demographics and the major clinical intervention data of the injured children (*n* = 969)

		Total		TR		SYR		CUN		*p*
		Mean		*n* = 517, 53.35%		*n* = 371, 32.71%		*n* = 81, 8.36%		
Age		9.68 ± 5.38		10.5 ± 5.33		9.08 ± 5.27		7.14 ± 5.11		**< 0.0001**
Admission time (days)		2.52 ± 1.59		2.47 ± 1.61		2.56 ± 1.59		2.67 ± 1.47		0.1393
		*n*	%	*n*	%	*n*	%	*n*	%	
Gender	Female	447	46.13	230	23.74	168	17.34	49	5.06	**0.0249**
	Male	522	53.87	287	29.62	203	20.95	32	3.30	
Fracture Surgery	Performed	78	8.05	45	4.64	27	3.41	0	0.00	**0.0209**
	Not Performed	891	91.95	472	48.71	338	34.88	81	8.36	
Fasciotomy	Performed	168	17.34	111	11.46	50	5.16	7	0.72	**0.0006**
	Not Performed	801	82.66	406	41.90	321	33.13	74	7.64	
Amputation	Performed	35	3.61	21	2.17	13	1.34	1	0.10	0.4435
	Not Performed	934	96.39	496	51.19	358	36.95	80	8.26	
Discharge	Treated	588	60.68	295	30.44	228	23.53	65	6.71	0.0746
	Hospitalized	167	17.23	108	11.15	56	5.78	3	0.31	
	Transferred	188	19.40	99	10.22	77	7.95	12	1.24	
	Deaths	26	2.68	15	1.55	10	1.03	1	0.10	

TR: Turkish nationality, SYR: Syrian nationality, CUN: children of unknown nationality

The mean age of the cases who underwent fracture surgery, fasciotomy, and amputation were 10.53 ± 4.81, 9.96 ± 4.91, and 11.06 ± 5.22, respectively. All amputations performed were primary; there were no traumatic amputations admitted or secondary amputations performed.

The cases with findings indicating compartment syndrome (*n* = 256) were not delayed, and fasciotomy was performed within the first eight hours upon diagnosis (*n* = 168, 17.34%).

In terms of the daily distribution of admissions based on the type of musculoskeletal injuries, the admissions of children with foot fractures in the first days were significantly increased (*p* = 0.0134). As the days progressed, admissions of children with pelvic fractures increased significantly (*p* = 0.0135). Regarding surgery rates, the fracture surgery and fasciotomy rates were significantly higher in cases admitted earlier (*p* < 0.0001 and *p* = 0.0009, respectively) (Table 2).

**Table 2:** The locations and the number of fractures and major surgeries

	Upper Limb			Lower Limb			Pelvis	*p*	
	Humerus	Radius/Ulna	Hand	Femur	Tibia/Fibula	Foot		Age	Days of admission
	*n*			*n*			*n*		
Fractures	51	34	7	48	39	9	9	0.06071	0.5811
Fractures Operated	22	4	-	28	15	-	9	0.1901	**< 0.0001**
Fasciotomies	68			108			-	0.5469	**0.0009**
	**Transhumeral**	**Forearm**	**Wrist/Hand**	**Transfemoral**	**Transtibial**	**Ankle/Foot**	**Hip**		
Amputations	4	3	3	5	8	12	-	0.1033	0.1963

There were no differences between the mean age of the cases based on the locations and the type of treatment of the fractures (*p* > 0.05).

With regard to nationality, there were no significant differences in the date of admission, the number of cases that underwent amputation, and the number and type of discharges. However, the results involving age showed substantial differences between the groups in terms of nationality. The p values of the comparisons between Turkish, Syrian, and the CUN were as follows: Turkish vs. Syrian; *p* < 0.0001, Syrian vs. CUN; *p* = 0.0033, and Turkish vs. CUN; *p* < 0.0001).

The comparison of the number of cases who underwent fracture surgeries revealed significant differences between CUN and the other two groups (CUN vs. Turkish children; *p* = 0.0058, and CUN vs. Syrian children; *p* = 0.0054). Regarding the number of fasciotomy procedures, Turkish children differed greatly from the Syrian and CUN (*p* = 0.0018 and *p* = 0.0064, respectively).

The correlation analysis conducted using the data resulted in only one positive link, indicating that among CUN, older children were admitted later in the week between (the age and the admission day after the quakes among CUN, *r* = 0.443, *p* < 0.0001).

## Discussion

We aimed to provide a general overview of admission and the initial orthopedic surgeries conducted for musculoskeletal injuries in children admitted to the state hospitals in the Adana metropolitan area during the first week following the earthquake and to investigate the differences between the groups according to nationality.

The chaotic conditions that developed immediately after the first quake were greatly exacerbated by the second quake nine hours later. The emergency department of the hospitals was crowded, and the identification of the cases, which was considered to be the initial step, became extremely difficult. Zones in the hospitals, such as meeting rooms and halls, were converted into care stations for children in good clinical condition. Non-medical care was provided by the social services staff, and further identification methods, including DNA analysis, were carried out, and the children were gradually returned to their families.

The sudden increase in emergency room admissions was the initial challenge faced after the earthquake. Previous research has shown that the patient density in emergency rooms can increase up to 66% within the first 24 h following an earthquake [13, 14]. Regarding orthopedics, the records show that the peak daily admissions of cases requiring orthopedical intervention occurred on the second day. The total number of admissions in need of orthopedical care observed in the first three days represented 79.46% of the total. Thus, under similar disaster conditions trauma centers should expect to encounter the great majority of the cases in the first three days. Studies highlight that the initial days following a major earthquake are the most crucial days for healthcare facilities since the highest number of admissions and the lowest level of support from the authorities and non-profit organizations are anticipated [15, 16].

Jacquet et al., in their review on children with earthquake-related injuries, reported that fractures were the most common injury, ranging between 18.1% and 55.2%, and frequently occurred in the extremities [11]. The report on the Gujarat earthquake stated that the fracture rate of 1248 injury cases, of whom were children, was 51%. The rate in our study was slightly lower as 20.33%, yet the distribution according to the site of fracture was in parallel with the literature, having most commonly occurred at extremities, followed by the pelvis.

The analysis considering the orthopedical approach showed that there were 246 (28.99%) cases requiring major orthopedical surgery, which was slightly lower than the numbers of Bar-On et al. The authors stated that children presented with much higher rates of fractures, particularly in the femur, and the rate of surgery was 44% [17]. Similarly, MacKenzie et al., in their review, presented that the combined rate of major surgeries, including open reduction and internal fixation, external fixation, and amputation in the treatment of earthquake-related injuries was approximately 43.5% [18]. The comparison of the number of fracture surgeries performed between the nationality groups showed that the rate of CUN was lower than in the other two groups. There were no cases that underwent fracture surgery in CUN, whereas the rate of Syrian children was nearly half compared to Turkish patients (4.64% vs. 8.05%).

Tang et al. stated that crush injuries were one of the most common injuries occurring in an earthquake [19]. Gök and Melik reported that 265 patients were admitted to the emergency department in the first seven days after the earthquake, and 32 (28.5%) were diagnosed with acute compartment syndrome. They also highlighted that fasciotomy was performed on 43 extremities [5 (11.6%) upper and 38 (88.4%) lower] of these patients, and 16 (50%) of the fasciotomy patients were operated on in the emergency department [20]. The rate of crush injury cases in our study was 26.42% (*n* = 256), and 168 fasciotomies were performed. The overall rate of fasciotomies was 17.34%, whereas the rate was 64.29% when considered only for the cases with crush injuries, with the lower limb being the most common limb operated for fasciotomies. Hatamizadeh et al., presenting a slightly lower rate of 12.6%, reported that lower limb fasciotomies were performed on 14 of the 15 children among the 119 admitted after the Bam, Iran earthquake [21].

Selçuk et al., in their report studying the outcomes of the same earthquake as ours, stated that out of 26 children admitted presenting with crush syndrome, 5 (19.23%) required limb amputations [22]. Another study by Sarı et al. conducted on the cases of the same quake zone showed that the number of amputations performed on children was 7 (5.38%) [23]. Our findings regarding amputations were slightly lower (*n* = 35). The percentage of amputations according to the total number of children requiring orthopedic intervention was 3.61%; however, when compared to children with crush injuries, the rate was 13.67%.

Furthermore, the duration of stay in the hospital of an earthquake-injured child, particularly the ones who underwent orthopedic surgery, is considerably increased [24]. Besides, Morelli et al. indicated that the mean hospitalization duration in children with earthquake injuries ranged between 1.4 and 10.7 days [25]. Our findings show that at the end of the first week after the quakes, 60.68% of the children were discharged (*n* = 588), indicating a similar rate to the reports.

The clinical findings of our study may have presented controversial results compared to the results of the analyses of the cases collected in earthquake foci or other level I trauma centers. We attribute the difference to the high rate of occurrence of severe cases at the foci and the accumulation of patients with critical medical conditions at the level I trauma centers since the most severe cases were immediately transferred to these centers. However, the rates are different when, as in our study, the data of the hospitals with level II and above trauma centers are analyzed in a single pool.

Moreover, the high admission rate of 371 Syrians (32.71%) was noteworthy. Although there are studies indicating that language and cultural barriers play a crucial part in accessing medical care, particularly in admissions to hospitals [26–28], the high admission rate shows that a high number of earthquake-injured displaced children in Adana had access to the hospitals and the treatments in the event of a major earthquake. In addition, the high rate of injury among Syrian children relative to the host population is striking, as the Syrian population in the region is up to about 8%, suggesting that the difference in admission rates compared to Turkish children should have been much lower. Although there should be more explanations, the high injury rates in Syrian children may be attributed mainly to the poor level of disaster awareness and knowledge and the housing and community conditions. Reports suggest that even residents of countries where disasters most frequently occur can be troubled when settled in other countries. Bhandari et al. stated that Nepalese who immigrated to Japan demonstrated poor levels of information and skills regarding geological disasters. The authors also noted that the barriers to accessing relevant information were also significant [29]. In terms of the conditions of accommodation, Türkoğlu and Elmastaş stated that 87.5% of displaced Syrians lived in detached houses in low-income neighborhoods [30].

Armenian et al. in a study involving 32.743 people suggested that the chance of getting injured was 1.8 times higher, a type of housing similar to the most displaced Syrian lived in [31]. Finally, although the admission rate of Turkish children was 53.35%, as the global numbers of UNHCR stating that more than 2 million children were born as refugees between 2018 and 2023, and the high rate of refugees settled in Adana province, we assume that the ratios in terms of nationality might require a slight correction in favor of the displaced [2]. Unfortunately, we did not have the resources to confirm the origin of the survivors.

### Limitations

The retrospective design of the study is one important limitation. In addition, there was too much missing information in the medical records from which we obtained the data for our analysis. Not to mention that the medical records kept at the beginning of the recovery from severe disasters are often incomplete. Nevertheless, we confirm that the data analyzed in our study is accurate. Finally, the generalization of the outcomes of the analysis may not be possible for all displaced children residing in the Adana province. Thus, overall interpretation requires attention. The injury overload exceeding the healthcare capacity of the metropolitan state hospitals caused by the severe earthquake, summed by the intense admission of unaccompanied children who were not capable of accurately providing medical and demographic information, limited the availability of precise data regarding the cases.

## Conclusion

The study has shown that, in the event of a severe earthquake, a level I trauma center in an earthquake zone with little or no physical damage had to take on a remarkably heavy workload, treating large numbers of severely injured patients in a short period of time. Despite the addition of cultural and language barriers accounting for approximately a third of the patients as obstacles in the delivery of healthcare under disaster conditions, our study has revealed that after the severe earthquake disaster, the city of Adana state hospitals have served to the best of their abilities and provided unbiased healthcare for all the children of the region. Regarding orthopedic care, in the event of an earthquake, most pediatric cases requiring major surgery should be sent to level I trauma centers, which are the most qualified in terms of staff and equipment. The authors emphasize the observation that in this particular calamity, the response was given accordingly, highlighting the awareness of the role that level I trauma centers should always be ready for such intensity due to the short preparation time in case of a severe disaster.

## Data Availability

The datasets used and/or analyzed during the current study are available from the corresponding author on reasonable request.
